# Financial Relationships Between Pharmaceutical Companies and Internal Medicine Societies

**DOI:** 10.1001/jamanetworkopen.2024.4777

**Published:** 2024-04-03

**Authors:** Anju Murayama, Kenichi Higuchi, Yuki Senoo

**Affiliations:** 1 Tohoku University School of Medicine, Sendai, Miyagi, Japan; 2 Department of Population Health Science and Policy, Icahn School of Medicine at Mount Sinai, New York, New York; 3 Higashi Totsuka Memorial Hospital, Yokohama, Kanagawa, Japan

## Abstract

This cross-sectional study uses payment data publicly disclosed by pharmaceutical companies affiliated with the Japan Pharmaceutical Manufacturers Association to describe their financial relationships with the subspecialty societies of the Japanese Society of Internal Medicine.

## Introduction

Professional medical societies play essential roles in delineating health care standards, serving a vital function in establishing and progressing the benchmarks for medical practice. Many societies receive funding from pharmaceutical companies to support their research, educational, and academic activities.^1^ However, financial relationships with pharmaceutical companies could introduce biases in decision-making and recommendations endorsed by societies.^2^ It is paramount for societies to manage financial relationships with pharmaceutical companies to preserve their independence and integrity.^3,4^ In Japan, substantial financial relationships exist between pharmaceutical companies and individual board members of internal medicine subspecialty societies of the Japanese Society of Internal Medicine (JSIM).^4^ However, information regarding the financial relationships between these societies and the pharmaceutical industry in Japan is limited.

## Methods

This cross-sectional study describes the financial relationships between pharmaceutical companies and all societies under JSIM by using payment data publicly disclosed by the companies affiliated with the Japan Pharmaceutical Manufacturers Association (JPMA). The JSIM encompasses 15 subspecialty societies, all of which were included in this study. The JPMA mandates that its member companies disclose their payments to these societies on each company’s website. Because this study was an analysis of publicly available data of nonhuman participants, institutional review board review was not required in accordance with Ethical Guidelines for Medical Research Involving Human Participants at Tohoku University. This study adhered to the Strengthening the Reporting of Observational Studies in Epidemiology (STROBE) guideline.

We extracted data on all payments made to the 15 societies between 2017 and 2020 by companies affiliated with the JPMA from the publicly accessible database^5^ and the company websites (for payments in 2021).^6^ Descriptive analyses of the payment data were conducted from December 2023 to February 2024. Payment amounts were converted to US dollars using the 2021 average monthly exchange rate of ¥109.795 per $1. All statistical analyses were performed using Excel, version 17.0 (Microsoft Corporation).

## Results

All 15 societies received payments from pharmaceutical companies between 2017 and 2021, totaling $86 167 759 for the 5 years (Table). The median (IQR) 5-year combined payment per society was $4 846 425 ($4 338 704-$7 047 076); mean (SD), $5 744 517 ($2 862 964). Notably, $65 411 727 (75.9%) of all payments to the societies were for cosponsored conferences and seminars. However, it was not possible to ascertain from the payment data whether the sponsor company’s drugs or products were advertised and/or discussed at these societies’ conferences.

**Table.** Payments From Pharmaceutical Companies to 15 Internal Medicine Subspecialty Societies in Japan

Variable	Payment, US $^a^
Total payment	86 167 759
5-y Combined payment per society	
Mean (SD)	5 744 517 (2 862 964)
Median (IQR)	4 846 425 (4 338 704-7 047 076)
Payments by category	
Expenses of cosponsored conferences and seminars, US $ (%)^b^	65 411 727 (75.9)
Donations to academic society, US $ (%)^c^	10 458 245 (12.1)
Other payments, US $ (%)^d^	6 745 257 (7.8)
General donation for unspecified purposes, US $ (%)^e^	3 552 532 (4.1)
Payments by year	
2017	17 546 051
2018	14 042 515
2019	19 242 034
2020	16 793 777
2021	18 543 383

^a^ Payment amounts were converted to US dollars using the 2021 average monthly exchange rate of ¥109.795 per $1.

^b^ Expenses of cosponsored conferences cover the fees for booth or space rental at societies’ conferences, seminars on companies’ products, and advertisement fees in the societies’ journals and conferences.

^c^ Designated for specific conferences or meetings of the professional medical societies.

^d^ Payments for societies’ research projects, educational programs, and paper awards sponsored by the companies.

^e^ Payments to the main body of professional medical societies without specifying the donation’s purpose.

Of the 15 societies for which data were obtained, the Japanese Society of Hematology received the highest payments, totaling $12.0 million (13.9% of all payments) from 2017 to 2021 (Figure). The next 4 highest payments were to the Japanese Circulation Society ($10.2 million [11.9%]), Japanese Society of Medical Oncology ($8.0 million [9.4%]), Japan Diabetes Society ($7.0 million [8.1%]), and Japanese Society of Gastroenterology ($7.0 million [8.1%]).

**Figure.**
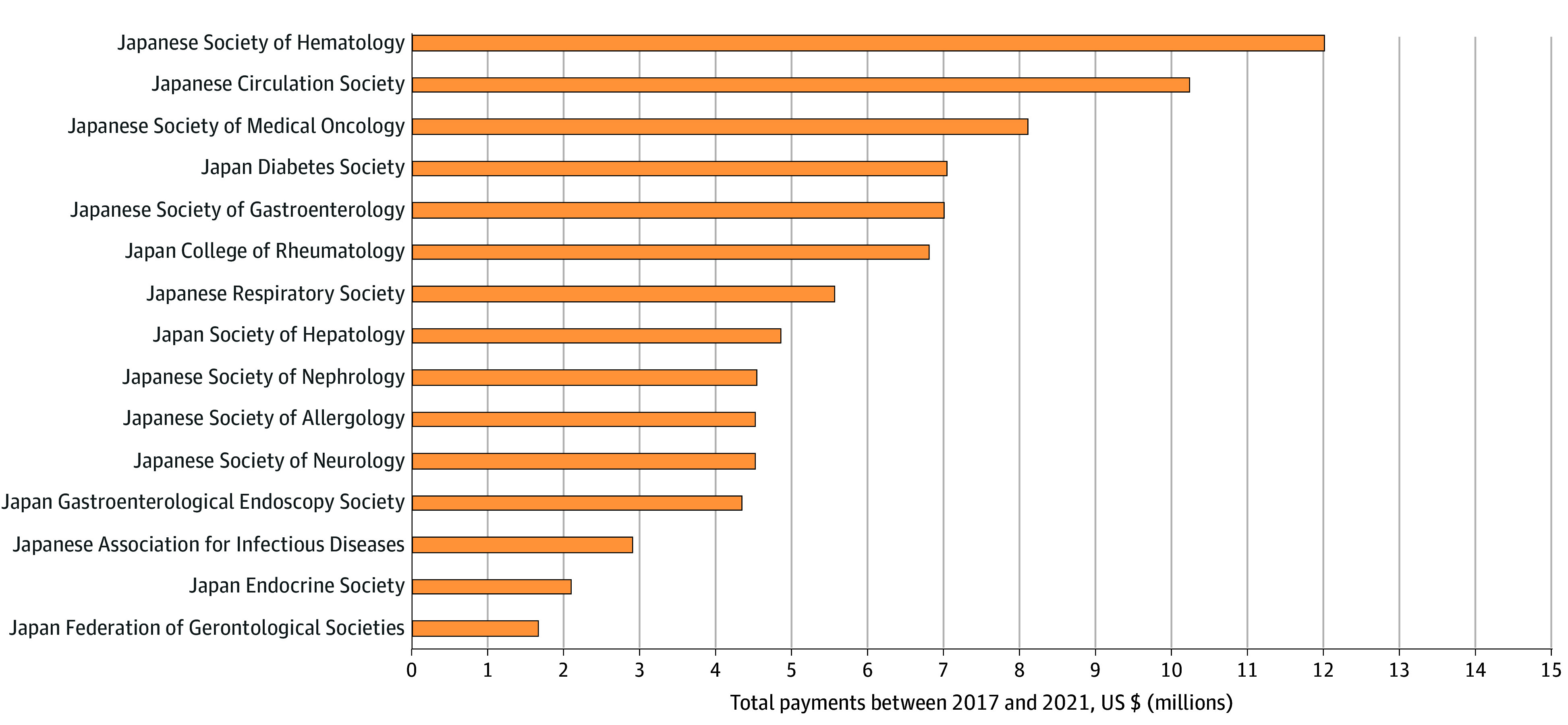
Payments by Pharmaceutical Companies to Japanese Medical Professional Societies, 2017 to 2021 Pharmaceutical payments were made to 15 Japanese medical societies in internal medicine subspecialties.

## Discussion

This cross-sectional study revealed that all 15 leading internal medicine subspecialty societies in Japan received a total of $86.1 million from 2017 to 2021. These payment amounts were more extensive than those reported for the 16 Royal Colleges in the United Kingdom (approximately $11 million from 2015 to 2022).^1^ In Japan, pharmaceutical companies are permitted to sponsor professional medical societies, assisting in organizing academic conferences where they sometimes present seminars on their products, known as luncheon seminars. Such relationships have raised concerns over potential conflicts of interest, leading to the JSIM discontinuing sponsorships from pharmaceutical companies after 2019.

This study has limitations, including potential inaccuracies in the payment data disclosed by companies and a lack of information on payments from companies not affiliated with the JPMA. Nevertheless, the financial ties between the pharmaceutical industry and these societies, which play a crucial role in developing clinical guidelines and certifying specialists in Japan, could pose a risk to the integrity and public trust in their recommendations and professional activities.

## Notes

Supplement.
Data Sharing Statement
