# Supplementary material for: Financial Relationships Between Pharmaceutical Companies and Internal Medicine Societies
Source: JAMA Netw Open. 2024 Apr 3;7(4):e244777. doi: 10.1001/jamanetworkopen.2024.4777 (PMC10993069; doi:10.1001/jamanetworkopen.2024.4777)
Supplement: Supplement. — Data Sharing Statement [file jamanetwopen-e244777-s001.pdf]

Data Sharing Statement

Murayama. Financial Relationships Between Pharmaceutical Companies and Internal Medicine Societies. *JAMA Netw Open*. Published April 03, 2024.
doi:10.1001/jamanetworkopen.2024.4777

Data
Data available: No

Additional Information
Explanation for why data not available: Due to the privacy protection, the dataset used in this study is available from the corresponding author upon reasonable request.
